# *Campylobacter* Infection in Poultry-Processing Workers, Virginia, USA, 2008–2011

**DOI:** 10.3201/eid1902.121147

**Published:** 2013-02

**Authors:** Marie A. de Perio, R. Todd Niemeier, Seth J. Levine, Karen Gruszynski, John D. Gibbins

**Affiliations:** Author affiliations: Centers for Disease Control and Prevention, Cincinnati, Ohio, USA (M.A. de Perio, R.T. Niemeier, J.D. Gibbins);; Virginia Department of Health, Richmond, Virginia, USA (S.J. Levine, K. Gruszynski)

**Keywords:** Campylobacter species, Campylobacter jejuni, Campylobacter coli, campylobacteriosis, occupational, employees, poultry, bacteria, humans, zoonoses, Virginia, gastrointestinal illness, poultry processing

## Abstract

During a health hazard evaluation, we investigated 29 cases of laboratory-diagnosed *Campylobacter* infection among workers at a poultry-processing plant. Most infected employees worked at the plant <1 month, worked as live hangers, and lived at a state-operated center. To lessen the infection risk, we recommended improvements to engineering and administrative controls at the plant.

*Campylobacter* spp., commensal organisms of poultry, are several common bacterial causes of gastrointestinal infection in the United States (*1*). *Campylobacter* infection, or campylobacteriosis, affects an estimated 2.4 million persons each year (*2*) and is most often associated with sporadic illness rather than outbreaks. Transmission typically occurs through consumption of undercooked poultry or handling of raw poultry (*3*,*4*). As part of a health hazard evaluation requested by plant management (*5*,*6*), we report a case series of laboratory-diagnosed *Campylobacter* infections among employees at a poultry-processing plant in Virginia during 2008–2011. As a public health response, according to Title 45 Code of Federal Regulations Part 46, this evaluation was determined not to require review by an institutional review board.

## The Study

During the period studied, the poultry plant processed 300,000–350,000 birds per day and employed ≈1,000 persons who worked in 2 main processing areas: first processing and second processing. In first processing, birds were unloaded, shackled (in an area called live hang), stunned, killed, scalded, defeathered, eviscerated, and chilled. In second processing, carcasses were rehung, washed, cooled, and packaged. The plant was under the regulatory authority of the Food Safety and Inspection Service of the US Department of Agriculture. At any given time, the plant employed 24–35 persons who were residents of 1 of 2 local diversion centers (i.e., participants of a 16- to 20-week residential work assignment program operated by the Virginia Department of Corrections). The plant had a medical office, with limited diagnostic capabilities, staffed by licensed practical nurses. Employees requiring additional medical evaluation were referred to outside providers.

Using occupation data in Virginia Department of Health case reports and Virginia Department of Corrections records, we identified persons who had laboratory-diagnosed *Campylobacter* infection while employed at the plant during January 2008–May 2011. To capture all possible cases, we defined a case-patient as a plant employee with *Campylobacter* infection diagnosed by culture or enzyme immunoassay. We reviewed case-patient records from the Virginia Department of Health, Virginia Department of Corrections, and local medical providers and obtained additional work history information from the plant.

To determine the background incidence of reported gastrointestinal illness among plant employees, we reviewed encounter records (for January 2010–September 2011) from the plant’s medical office. We categorized an encounter as gastrointestinal illness–related if the employee reported diarrhea, abdominal cramps, nausea, or vomiting without another reason listed, such as nausea related to pregnancy or migraine headaches. We then tabulated gastrointestinal illness–related encounters by month.

We identified 29 cases of laboratory-diagnosed *Campylobacter* infection during January 2008–May 2011 in persons employed at the poultry-processing plant. Of the 29 persons, 23 were infected with *C. jejuni*, 1 was infected with *C. coli*, and 5 were infected with an unspecified *Campylobacter* species. Twenty-seven cases were diagnosed by stool culture; 2 were diagnosed by stool enzyme immunoassay.

The median age of case-patients was 29 years (range 19–52 years); 28 (97%) were men. Twenty-six (90%) case-patients were residents of a diversion center, and 3 lived at a private residence. Of the 29 case-patients, 27 (93%) worked in first-processing areas, including the live-hang (n = 18), evisceration (n = 8), and kill (n = 1) rooms, and 2 worked in second-processing areas, including the rehang (n = 1) and cut-up (n = 1) rooms. Twenty-four (83%) case-patients worked at the plant for <1 month before illness onset.

**Figure 1:**
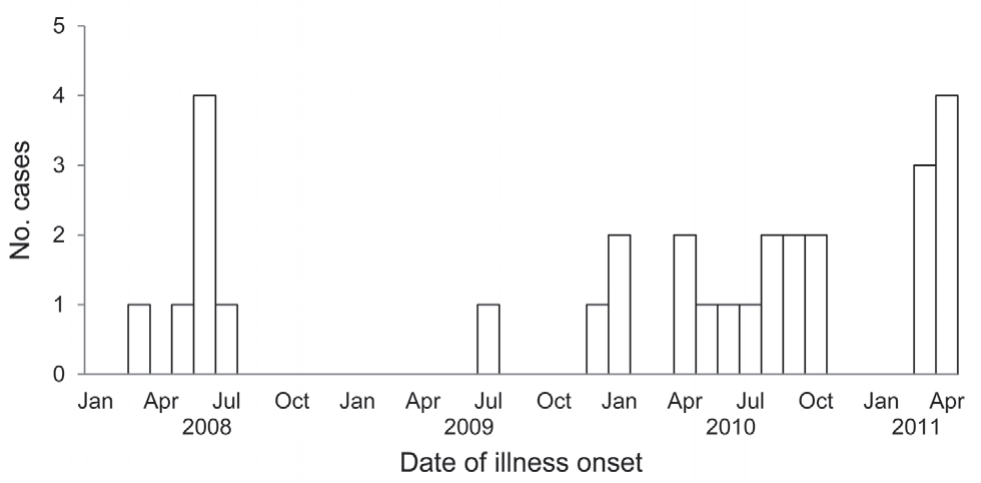
Number of laboratory-diagnosed *Campylobacter* infections, by month of illness onset, in employees at a poultry-processing plant, Virginia, USA, 2008–2011.

We obtained medical records for 24 case-patients, 3 of whom reported having been sought care in the plant medical office. These 24 case-patients were all reported to have had diarrhea while sick. Other signs and symptoms included abdominal cramping (n = 14), fever (n = 9), nausea and vomiting (n = 6), headache (n = 7), and muscle aches (n = 3). Of the 29 case-patients, 1 was hospitalized; there were no deaths. Figure 1 shows the number of cases by month of symptom onset.

**Figure 2:**
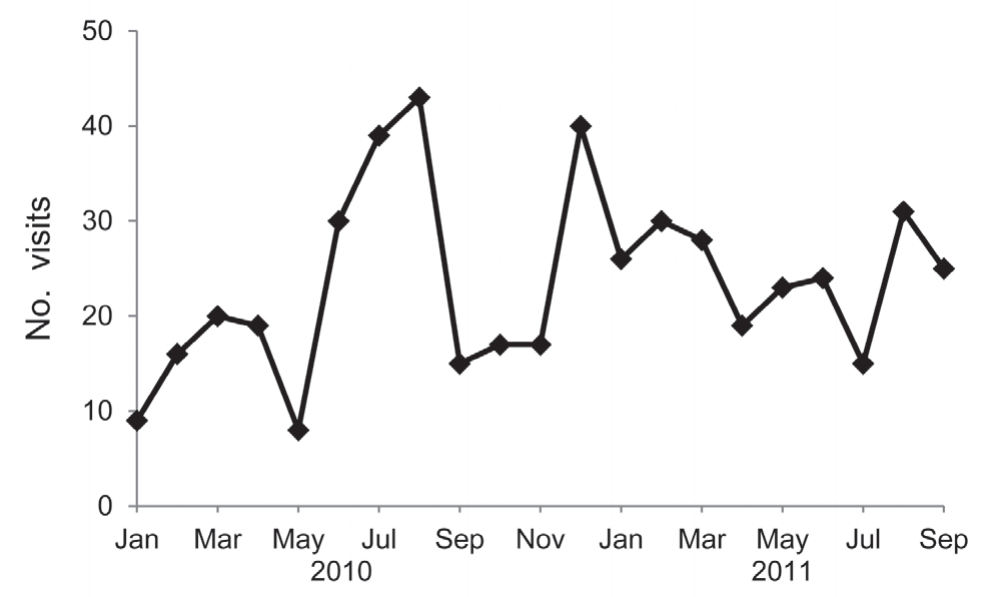
Number of gastrointestinal illness–related visits to the medical office in a poultry-processing plant, Virginia, USA, 2008–2011.

In 2010, a total of 1,716 encounters at the plant’s medical office were recorded; 273 (16%) were associated with gastrointestinal symptoms. During January 2011–September 2011, a total of 1,543 encounters at the plant’s medical office were recorded, of which 221 (15%) were related to gastrointestinal symptoms (Figure 2). Multiple peaks of visits for gastrointestinal illness were seen during summer 2010 and winter 2010–11, and a smaller peak occurred in summer 2011. Most other reasons for plant medical office visits were for injury reporting, first aid, and musculoskeletal symptoms.

## Conclusions

Our investigation revealed 29 cases of laboratory-diagnosed *Campylobacter* infection in employees at the poultry-processing plant during January 2008–May 2011. Most (62%) cases occurred among employees in the live-hang area who are responsible for lifting live chickens from the supply conveyer and hanging them by their feet from a shackle conveyor. This area has a known high potential for contamination with *Campylobacter* spp. because the feathers, skin, crop, cloaca, and feces of birds brought to slaughter are often highly contaminated with *Campylobacter* spp. (*7*,*8*). Only ≈50 of the ≈1,000 employees work in the live-hang area, suggesting that these employees are disproportionately infected with *Campylobacter* spp. Preharvest practices by the plant and hatcheries may not be sufficient for controlling *Campylobacter* spp. contamination of live birds. The US Department of Agriculture has noted that high bacterial loads of *Campylobacter* spp. on live birds can undermine other in-plant interventions (*9*).

All but 3 case-patients were residents of a diversion center. Many diversion center residents are assigned to work in the live-hang area. Most (83%) case-patients had worked at the plant for <1 month before illness onset. Our finding of illness in new employees is similar to findings from previous investigations of poultry workers. For example, an outbreak investigation of *Campylobacter* infection among poultry workers in Sweden revealed that infection attack rates among inexperienced teenage holiday workers were higher than those among experienced staff (*10*). Another study found that levels of antibodies to *Campylobacter* spp. in long-term workers (employed >1 month) in Sweden were significantly higher than levels in short-term workers (employed <1 month) and in blood donors with no special exposure to poultry (*9*). Those findings indicate that for poultry workers, the highest risk for work-related *Campylobacter* infection is during the first weeks of work, after which the workers develop immunity that may be protective against future symptomatic infection (*11*).

In our investigation, the apparent overrepresentation of diversion center residents among employees with *Campylobacter* infection may be partially attributed to their better access to health care compared with access by permanent employees. Approximately 15% of ≈3,000 encounters at the plant’s medical office during January 2010–September 2011 were related to gastrointestinal disorders. The numbers of cases of *Campylobacter* infection and gastrointestinal illness that we found among plant employees are likely an underestimation of the true numbers. This may be due to an unwillingness to report illness because of the plant’s lack of paid sick leave and employees’ difficulty in accessing medical care.

On the basis of our findings, we recommended that plant management strengthen efforts to reduce *Campylobacter* contamination, particularly in the live-hang area. Efforts should incorporate engineering controls, such as improved sanitation, ventilation system modifications, and installation of hands-free soap dispensers and waste receptacles. We also recommended that employee training (in English and Spanish) and compliance with plant policies related to hand hygiene and the use of personal protective equipment be improved, especially among temporary employees. Poultry-processing plants should regularly review their illness records and work with local health departments to ensure that all cases and outbreaks of *Campylobacter* infection are reported.
